# Supplementary material for: Mutation in Hemagglutinin Antigenic Sites in Influenza A pH1N1 Viruses from 2015–2019 in the United States Mountain West, Europe, and the Northern Hemisphere
Source: Genes (Basel). 2022 May 19;13(5):909. doi: 10.3390/genes13050909 (PMC9141826; doi:10.3390/genes13050909)
Supplement: Supplementary file 1 [file genes-13-00909-s001.zip › Figure S11_Supplementary_Figure_circos.pdf]

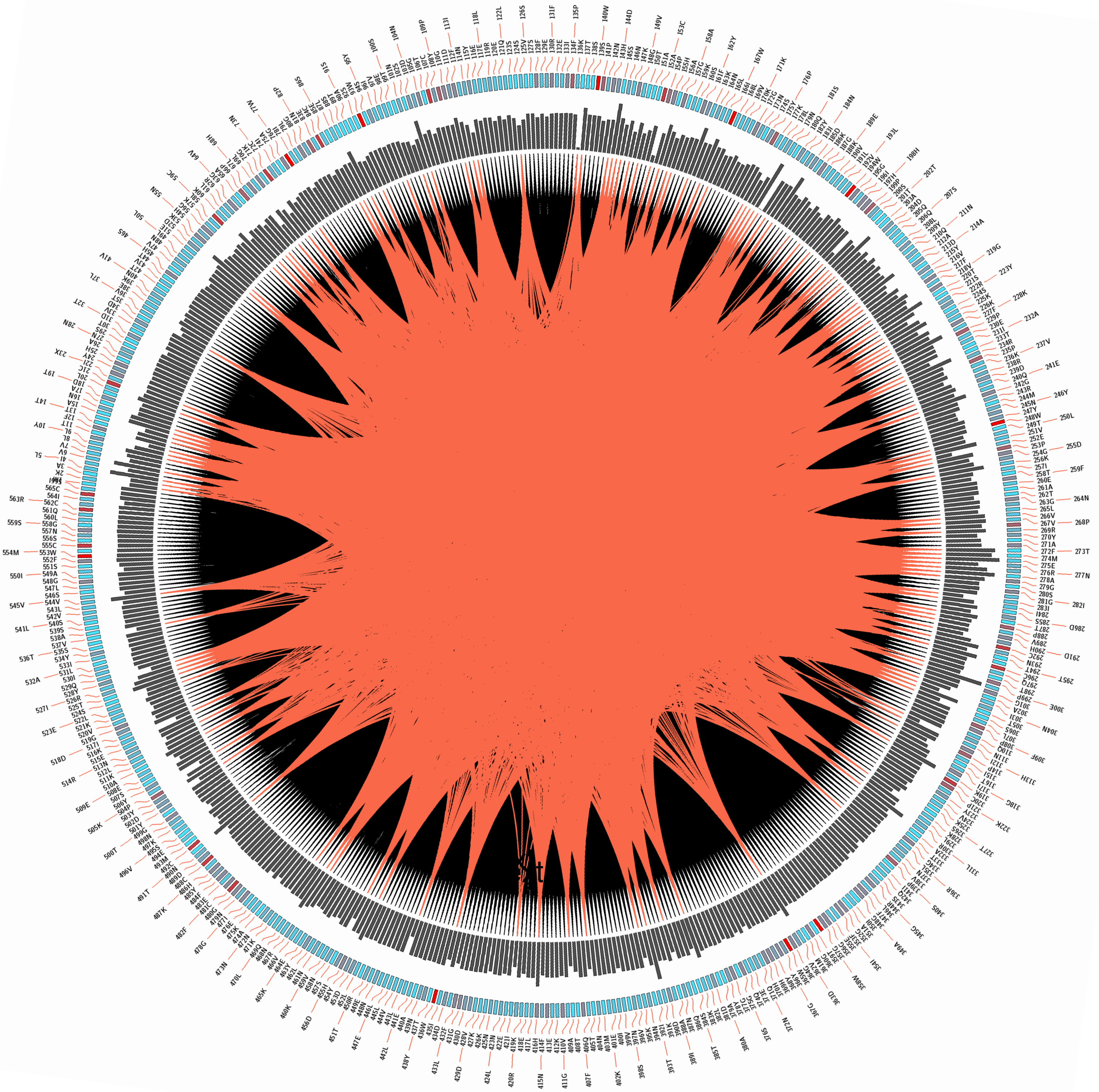

Co-evolution Circos Plot of Northern Hemisphere HA Sequences. A graphical view showing the co-evolution between amino acid positions in HA sequences. The outermost circle displays the amino acid residue number along with the reference sequence. The multicolored boxes display the highest conserved MSA positions in red with the less conserved MSA positions in blue. Proximity Mutual Information (MI) is presented as a histogram in the third circle from the edge, and cumu-lative MI is similarly represented in the fourth circle. Lines in the center link pairs of amino acid positions with MI >6.5, with the red lines indicating the top 5% with black lines being the other 95%.
