# Supplementary material for: Mutation in Hemagglutinin Antigenic Sites in Influenza A pH1N1 Viruses from 2015–2019 in the United States Mountain West, Europe, and the Northern Hemisphere
Source: Genes (Basel). 2022 May 19;13(5):909. doi: 10.3390/genes13050909 (PMC9141826; doi:10.3390/genes13050909)

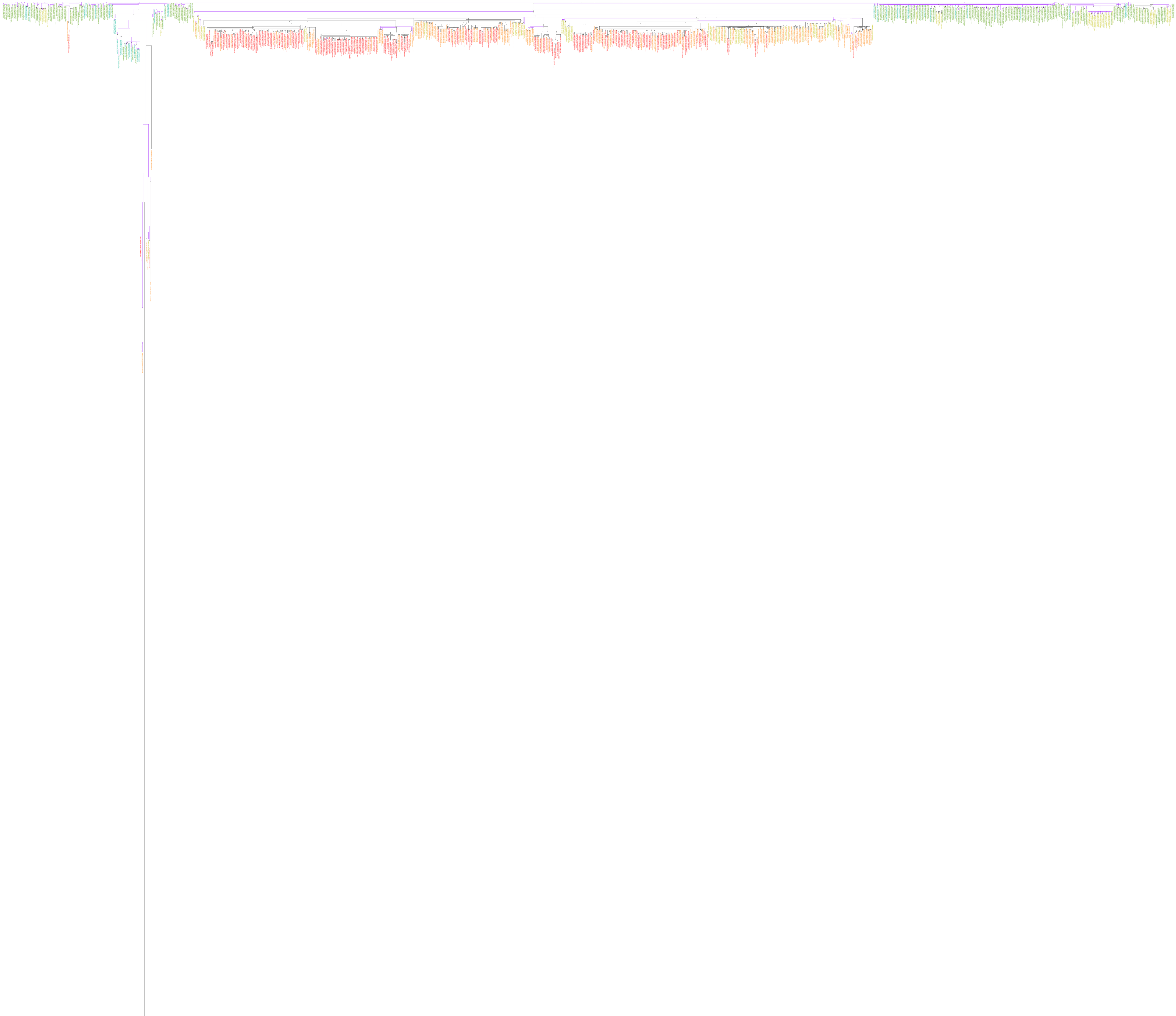

Supplement: Supplementary file 1 [file Figure S3_Supplementary_Figure_USA-RAxML.pdf › Figure S3_Supplementary_Figure_USA-RAxML.pdf]
